# Dr. Harry Bolton Ransom

**Published:** 1915-01

**Authors:** 


**Dr. Harry Bolton Ransom,** a graduate of the Botanic Medical Society of Connecticut and of the Metropolitan Medical School of New York City, died at his home, 172 Breckenridge Street, Buffalo, November 26, 1914. He was born in Clarence Hollow, November 20, 1832, practiced for some time on Grand Island, was supervisor from his town for several years, a member of the N. Y. Assembly in the early '70's, and was coroner of Erie County 1884-8.

